# S206. KNOWLEDGE ABOUT CAUSES OF RELAPSE DURING PSYCHOEDUCATION IN PATIENTS LIVING WITH SCHIZOPHRENIA- A QUALITATIVE ANALYSIS

**DOI:** 10.1093/schbul/sby018.993

**Published:** 2018-04-01

**Authors:** Anoop Gupta, Mamidipalli Spoorthy, Bina Gurung, Robin Jha, Bishnu Acharya

**Affiliations:** 1 National Medical College; 2 All India Institute of Medical Sciences, Raipur

## Abstract

**Background:**

Evidence till date shows different reasons of relapse in schizophrenia around the world. However, there was almost no reliable data from Nepal. We want to report a thematic study based on the reports of 12 patients living with schizophrenia and their family members. These patients were approached during psychoeducation group sessions.

**Methods:**

Twelve patients with a diagnosis of schizophrenia as per Diagnostic and Statistical Manual of mental disorders-5 criteria, who were accompanied by their family members were selected. A minimum duration of illness of 5 years was required as inclusion criteria. Semi structured interviews were conducted with patient and family members separately in 1–2 sessions. Questions were mainly related to their knowledge about causes of relapse in patients in their perspective. Interviews were recorded and transcripts were generated. All the transcripts were read separately by the 3 investigators and common themes agreed upon by all the investigators were generated. We used content analysis for the purpose of the study. A total of 36 sessions psychoeducation were taken in in-patients from National Medical College, Birgunj, Nepal. Eight out of 12 patients were males. The group therapy was psycho-education oriented and based on NIMHANS manual for family-based intervention in schizophrenia. We included those patients who were admitted and improving as per PANSS score (more than 50% of the score at admission).

**Results:**

The patients’ family members told that these sessions were useful because their issues were discussed and addressed and simpler terms were used during the process. The patients showed ability to participate and understand the proceedings though not always. Two of the patients had sub-normal intelligence and so they were not benefited more than being heard about their sufferings. Their family members reported a better understanding of the illness and non-pharmacological approach for these patients after the sessions. Participants were encouraged to make notes out of the discussions in the sessions but few of them did so.

Following themes emerged after the analysis of transcribed verbatim from the patients and family members.

Themes generated from patient’s versions:

1. Residual negative/depressive symptoms

2. Critical comments from family members

3. Adverse effects of medications

4. Improper education about the duration of treatment

Themes generated from family member’s versions:

5. Lack of awareness about the illness

6. Belief in super natural causes

7. Affordability issues

8. Poor insight about the illness

9. Poor compliance to medications

10. Stress

**Discussion:**

Conclusion: Educating our patients can be tiring and mundane during regular out-patient department. However, the psychoeducation sessions are very important part of the treatment. During that process we should anticipate the possible causes of relapse and educate the same for better outcome.

